# Gut Microbiome Alterations Associated with Diabetes in Mexican Americans in South Texas

**DOI:** 10.1128/msystems.00033-22

**Published:** 2022-04-28

**Authors:** Suet-Ying Kwan, Caroline M. Sabotta, Aron Joon, Peng Wei, Lauren E. Petty, Jennifer E. Below, Xiaogang Wu, Jianhua Zhang, Robert R. Jenq, Ernest T. Hawk, Joseph B. McCormick, Susan P. Fisher-Hoch, Laura Beretta

**Affiliations:** a Department of Molecular and Cellular Oncology, The University of Texas MD Anderson Cancer Center, Houston, Texas, USA; b Department of Biostatistics, The University of Texas MD Anderson Cancer Center, Houston, Texas, USA; c Vanderbilt Genetics Institute and Department of Genetic Medicine, Vanderbilt University Medical Center, Nashville, Tennessee, USA; d Department of Genomic Medicine, The University of Texas MD Anderson Cancer Center, Houston, Texas, USA; e Department of Clinical Cancer Prevention, The University of Texas MD Anderson Cancer Center, Houston, Texas, USA; f School of Public Health, University of Texas Health Science Center at Houston, Brownsville Regional Campus, Brownsville, Texas, USA; University of California, San Francisco

**Keywords:** gut microbiome, metagenome, diabetes, health disparity, Mexican American population, diabetes

## Abstract

Mexican Americans have a high prevalence of diabetes and burden of diabetes-related complications, highlighting the need for novel preventive strategies and noninvasive predictors of diabetes risk tailored to this population. Changes in the gut microbiome have the potential to predict diabetes. Here, we aimed to identify alterations in the gut microbiome associated with diabetes in the high-risk population of Mexican Americans in South Texas. Stool samples were collected from 216 subjects from the population-based Cameron County Hispanic Cohort. Among them, 75 had type 2 diabetes. Taxonomic and functional profiling of the stool samples were assessed by 16S and shotgun metagenomic sequencing, and the influence of genetic factors was explored. The gut microbiome of subjects with diabetes was enriched with proinflammatory Proteobacteria members (Enterobacteriaceae, Escherichia*-*Shigella) and depleted of butyrate-producing Clostridiales members (Faecalibacterium prausnitzii, Peptostreptococcaceae, and Clostridium *sensu stricto* 1). The accompanying metagenomic changes in subjects with diabetes suggested dysregulated amino acid metabolism, reduced galacturonate and glucuronate catabolism (correlating with Faecalibacterium prausnitzii abundance), and enriched heme biosynthesis (correlating with *Enterobacteriaceae* abundance). Polymorphism rs7129790 near *MMP27* was strongly associated with high *Proteobacteria* abundance and was more frequent in this cohort and in individuals of Mexican ancestry than in Europeans. In conclusion, Mexican Americans in South Texas with diabetes display distinct gut microbiome and metagenomic signatures. These signatures may have utility in risk modeling and disease prevention in this high-risk population.

**IMPORTANCE** The gut microbiome composition varies across ethnicities and geographical locations, yet studies on diabetes-associated microbiome changes specific to high-risk Mexican Americans are lacking. Here, we aimed to identify specific alterations associated with diabetes in this population, as well as host genetic factors that may explain increased disease susceptibility in this ethnic group. Using samples from a population-based cohort of Mexican Americans with a high prevalence of obesity and diabetes, we confirmed findings from studies on other ethnicities that suggested promotion of a chronic proinflammatory environment, loss of butyrate production, and compromised intestinal barrier integrity. High abundance of proinflammatory *Proteobacteria* was associated with a polymorphism that was more frequent in this cohort and in individuals of Mexican ancestry than in Europeans. Validation of microbiome-based risk models for diabetes should be evaluated in prospective cohort studies.

## INTRODUCTION

Hispanics are the largest ethnic minority in the United States and are disproportionately affected by diabetes, with the prevalence of diagnosed diabetes being 12.5% compared to 7.5% in non-Hispanic whites. Among adult Hispanics, those of Mexican origin have the highest prevalence at 14.4% (1). Furthermore, Hispanics have a higher burden of diabetes-related complications, including poor glycemic control, nephropathy, and retinopathy (1, 2). The presence of diabetes also significantly increases the risk of developing nonalcoholic fatty liver disease (NAFLD) and progression to nonalcoholic steatohepatitis (NASH), liver fibrosis, and cirrhosis (3). Due to the obesity and diabetes epidemics, the incidences of NASH and liver fibrosis are rising in the United States (4, 5). Innovative preventive strategies and noninvasive methods to identify those at high risk of diabetes, tailored to this population, are therefore urgently needed.

Gut microbiome profiles have the potential to complement host factors in predicting clinical outcomes. It has been recently demonstrated that taxonomic composition explains a significant amount of variance in clinical parameters, including body mass index (BMI), fasting blood glucose, and glycemic status, even after accounting for age, gender, diet, and host genetics (6). The gut microbiome composition is subject to significant variability across ethnicities and geographical locations (7, 8), yet studies on diabetes-associated microbiome changes specific to high-risk Hispanic populations are lacking.

Therefore, the aim of this study was to identify alterations in the gut microbiome associated with diabetes in the high-risk population of Mexican Americans in South Texas. To that end, we enrolled subjects from the Cameron County Hispanic Cohort (CCHC), a large population-based Mexican American cohort in South Texas with very high prevalences of diabetes (28%), obesity (51%), and chronic liver injury (39%) (9–11). We also aimed to identify possible genetic factors contributing to these microbiome changes, as well as functional changes in the gut microbiome.

## RESULTS

### Study population and stool taxonomic composition.

Stool samples were collected from 216 randomly selected subjects from the CCHC (Table 1). Among them, 118 (54.6%) were obese and 75 (34.7%) were diabetic. Subjects with diabetes were more likely to be born in Mexico (80.0% versus 63.1%; *P = *0.012), older (median of 57.0 versus 54.0 years; *P = *0.023), and had higher hemoglobin A1c (HbA1c) (7.6% versus 5.8%; *P < *0.001), insulin resistance as assessed by homeostatic model assessment (HOMA) scores (3.9 versus 2.2; *P < *0.001) and waist-to-hip ratios (1.0 to 0.9; *P < *0.001). They also had elevated circulating levels of alanine aminotransferase (ALT) (32.5 versus 26.0; *P = *0.012), alkaline phosphatase (95.0 versus 86.0 U/L; *P = *0.014), fasting glucose (136.0 versus 92.5 mg/dL; *P < *0.001), and triglycerides (143.0 versus 124.0 mg/dL; *P = *0.044), but lower low-density lipoprotein (LDL) cholesterol (96.0 versus 108.0 mg/dL; *P = *0.024). Among the 75 diabetic subjects, 46 had information on age at time of diagnosis, with a median of 49 years old (range of 20 to 67 years old).

**TABLE 1:** Demographic and clinical parameters of study participants with and without diabetes^*a*^

Parameter	Group^*b*^		*P*
	No diabetes (*n* = 141)	Diabetes (*n* = 75)	
Country of birth (*n* = 216)			
Mexico	89 (63.1%)	60 (80.0%)	0.012^*c*^
USA	51 (36.2%)	14 (18.7%)	
Other	1 (0.7%)	1 (1.3%)	
Male (*n* = 216)	38 (27.0%)	24 (32.0%)	0.435
Age (*n* = 216)	52.4 (19.0–89.0); 54.0	57.0 (34.0–81.0); 57.0	0.023^*c*^
BMI (kg/m^2^) (*n* = 215)	31.3 (18.8–49.0); 30.3	32.5 (20.9–50.0); 31.6	0.135
Obese (*n* = 215)	72 (51.4%)	45 (60.0%)	0.252
HbA1c (%) (*n* = 216)	5.8 (4.9–6.4); 5.8	8.2 (5.3–14.1); 7.6	<0.001^*c*^
Insulin (mIU/L) (*n* = 215)	12.4 (3.0–51.4); 10.1	11.9 (2.4–30.4); 10.8	0.933
HOMA (*n* = 214)	2.9 (0.6–12.3); 2.2	4.5 (0.5–11.8); 3.9	<0.001^*c*^
Waist circumference (cm) (*n* = 216)	103.3 (76.0–140.0); 104.0	107.3 (78.0–141.0); 105.0	0.059
Waist-to-hip ratio (*n* = 215)	0.9 (0.8–1.1); 0.9	1.0 (0.8–1.1); 1.0	<0.001^*c*^
Hypertension (*n* = 216)	42 (29.8%)	33 (44.0%)	0.051
Alcohol consumption (*n* = 216)			
Never	95 (67.4%)	49 (65.3%)	0.241
Moderate	42 (29.8%)	20 (26.7%)	
Heavy	4 (2.8%)	6 (8.0%)	
Smoking status (*n* = 216)			
Never	100 (70.9%)	48 (64.0%)	0.512
Former	32 (22.7%)	20 (26.7%)	
Current	9 (6.4%)	7 (9.3%)	
Blood tests			
AST (U/L) (*n* = 215)	20.7 (9.0–64.0); 19.0	21.8 (10.0–77.0); 19.0	0.642
Abnormal AST (*n* = 215)	6 (4.3%)	6 (8.1%)	0.348
ALT (U/L) (*n* = 215)	30.8 (15.0–114.0); 26.0	36.4 (12.0–128.0); 32.5	0.012^*c*^
Abnormal ALT (*n* = 215)	45 (31.9%)	34 (45.9%)	0.053
Total bilirubin (mg/dL) (*n* = 216)	0.5 (0.1–1.9); 0.5	0.5 (0.2–1.2); 0.5	0.689
Creatinine (mg/dL) (*n* = 216)	0.8 (0.4–1.4); 0.8	0.7 (0.4–1.8); 0.7	0.023^*c*^
Albumin (gm/dL) (*n* = 216)	3.9 (3.0–4.5); 3.9	3.9 (3.3–4.4); 3.9	0.997
Alkaline phosphatase (U/L) (*n* = 216)	88.5 (38.0–158.0); 86.0	97.6 (52.0–165.0); 95.0	0.014^*c*^
Fasting glucose (mg/dL) (*n* = 215)	93.4 (77.0–120.0); 92.5	159.6 (76.0–360.0); 136.0	<0.001^*c*^
Triglycerides (mg/dL) (*n* = 216)	140.7 (36.0–368.0); 124.0	197.1 (39.0–1596.0); 143.0	0.044^*c*^
Total cholesterol (mg/dL) (*n* = 215)	187.5 (50.0–303.0); 187.0	180.2 (77.0–274.0); 178.0	0.163
HDL cholesterol (mg/dL) (*n* = 216)	50.9 (30.0–109.0); 49.0	49.2 (27.0–84.0); 49.0	0.554
LDL cholesterol (mg/dL) (*n* = 210)	108.9 (33.0–196.0); 108.0	97.3 (8.0–187.0); 96.0	0.024^*c*^
Platelets (x10^9^/L) (*n* = 216)	257.0 (120.0–432.0); 249.0	251.2 (119.0–384.0); 249.0	0.720

a BMI, body mass index; HbA1c, hemoglobin A1c; HOMA, homeostatic model assessment; AST, aspartate aminotransferase; ALT, alanine aminotransferase; HDL, high-density lipoprotein; LDL, low-density lipoprotein.

b Data are presented as frequency (%) for categorical variables, or mean (range); median for continuous variables.

c Significant differences between the no-diabetes (*n* = 141) and diabetes (*n* = 75) groups (*P* < 0.05), as assessed by Fisher’s exact test for categorical variables and by the Mann-Whitney test for continuous variables.

**FIG 1:**
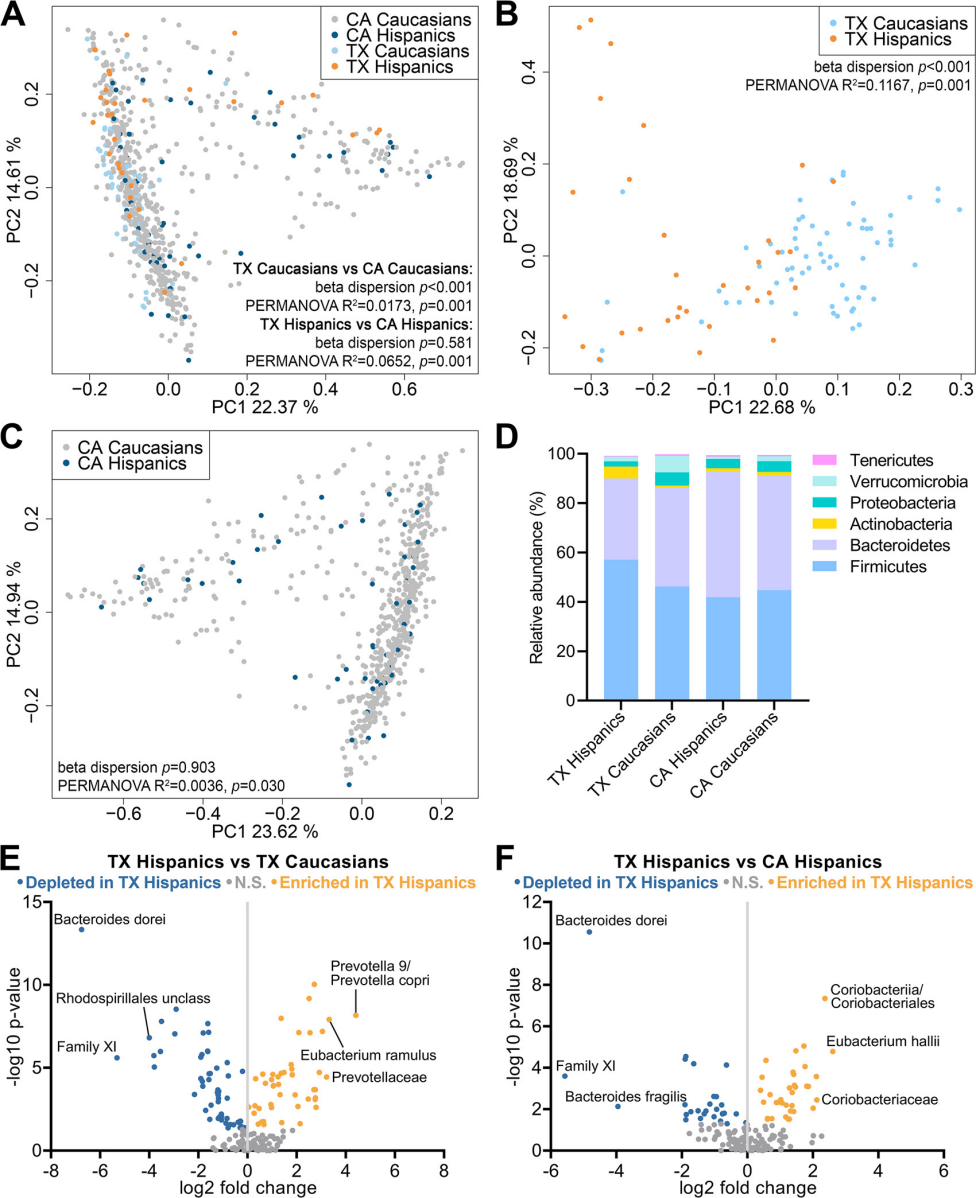
Impact of region and ethnicity on microbiome composition. Principal-coordinate analysis (PCoA) plots based on weighted UniFrac distances were generated for CA Caucasians, CA Hispanics, TX Caucasians and TX Hispanics (A); Caucasians and Hispanics in Texas (B); and Caucasians and Hispanics in California (C). Beta dispersion and permutational analysis of variance (PERMANOVA) test results comparing population groups are included in panels A to C. (D) Relative phylum abundance in each population group. (E and F) Volcano plots for differential bacterial abundance between TX Hispanics and TX Caucasians (E) and between TX Hispanics and CA Hispanics (F). Names are shown for the three most enriched and three most depleted taxa. Significance was determined by Mann-Whitney test. N.S., not significant.

To determine whether healthy Hispanics in Texas (TX Hispanics) had microbiome compositions that differed from those of healthy Caucasians in Texas (TX Caucasians) and from those of heathy Hispanics and Caucasians in California (CA Hispanics and CA Caucasians), we downloaded 16S stool sequencing data from 68 TX Caucasians, 636 CA Caucasians, and 51 CA Hispanics from the American Gut Project (AGP). For TX Hispanics, we included 31 healthy CCHC subjects that were not heavy drinkers and were without obesity, diabetes, abnormal aspartate aminotransferase (AST) levels, or abnormal ALT levels. Microbiome richness, measured by the number of observed operational taxonomic units (OTUs), was not significantly different between TX Hispanics and TX Caucasians, CA Hispanics, or CA Caucasians (see Fig. S1A in the supplemental material), nor between regions or ethnicities (Fig. S1A). Microbiome richness and evenness measured by the Shannon index was also not significantly different between TX Hispanics and the other three groups. However, Californians exhibited lower Shannon diversity than Texans (*P = *0.012) (Fig. S1B). In contrast, beta diversity analysis showed that the stool microbial composition of TX Hispanics was distinct from those of the other three groups (Fig. 1A). While both ethnicity and region had a significant impact, differences between Caucasians and Hispanics were stronger within Texas (beta dispersion *P < *0.001, permutational analysis of variance [PERMANOVA] *P = *0.001; 11.67% of variation explained) than within California (beta dispersion *P = *0.903, PERMANOVA *P = *0.030; 0.36% of variation explained) (Fig. 1B and C). TX Hispanics and CA Hispanics displayed similar degrees of heterogeneity but significantly different profiles (beta dispersion *P = *0.581, PERMANOVA *P = *0.001; 6.52% of variation explained) (Fig. 1A). At the phylum level, TX Hispanics had significant enrichment of Firmicutes and Actinobacteria and depletion of Bacteroidetes compared to the other three groups (Fig. 1D). The Prevotellaceae family, the Prevotella 9 genus, and Eubacterium ramulus were significantly enriched in TX Hispanics compared to TX Caucasians, while there was a significant depletion of the unclassified Rhodospirillales and Family XI families and of Bacteroides dorei (Fig. 1E). Compared to CA Hispanics, TX Hispanics were enriched in the Coriobacteria class, Coriobacteriaceae family, and Eubacterium hallii, and depleted in Family XI, Bacteroides dorei, and Bacteroides fragilis (Fig. 1F).

10.1128/msystems.00033-22.1 FIG S1 Alpha diversity comparison between regions and ethnicities. Number of observed operational taxonomic units (OTUs) (A) and Shannon index scores (B) among TX Hispanics compared to TX Caucasians, CA Hispanics, and CA Caucasians. Alpha diversity was also compared between regions and ethnicities. Significance was determined by Mann-Whitney test. Download FIG S1, TIF file, 1.2 MB. Copyright © 2022 Kwan et al. 2022 Kwan et al. https://creativecommons.org/licenses/by/4.0/ This content is distributed under the terms of the Creative Commons Attribution 4.0 International license.

### Microbiome signatures associated with diabetes.

**FIG 2:**
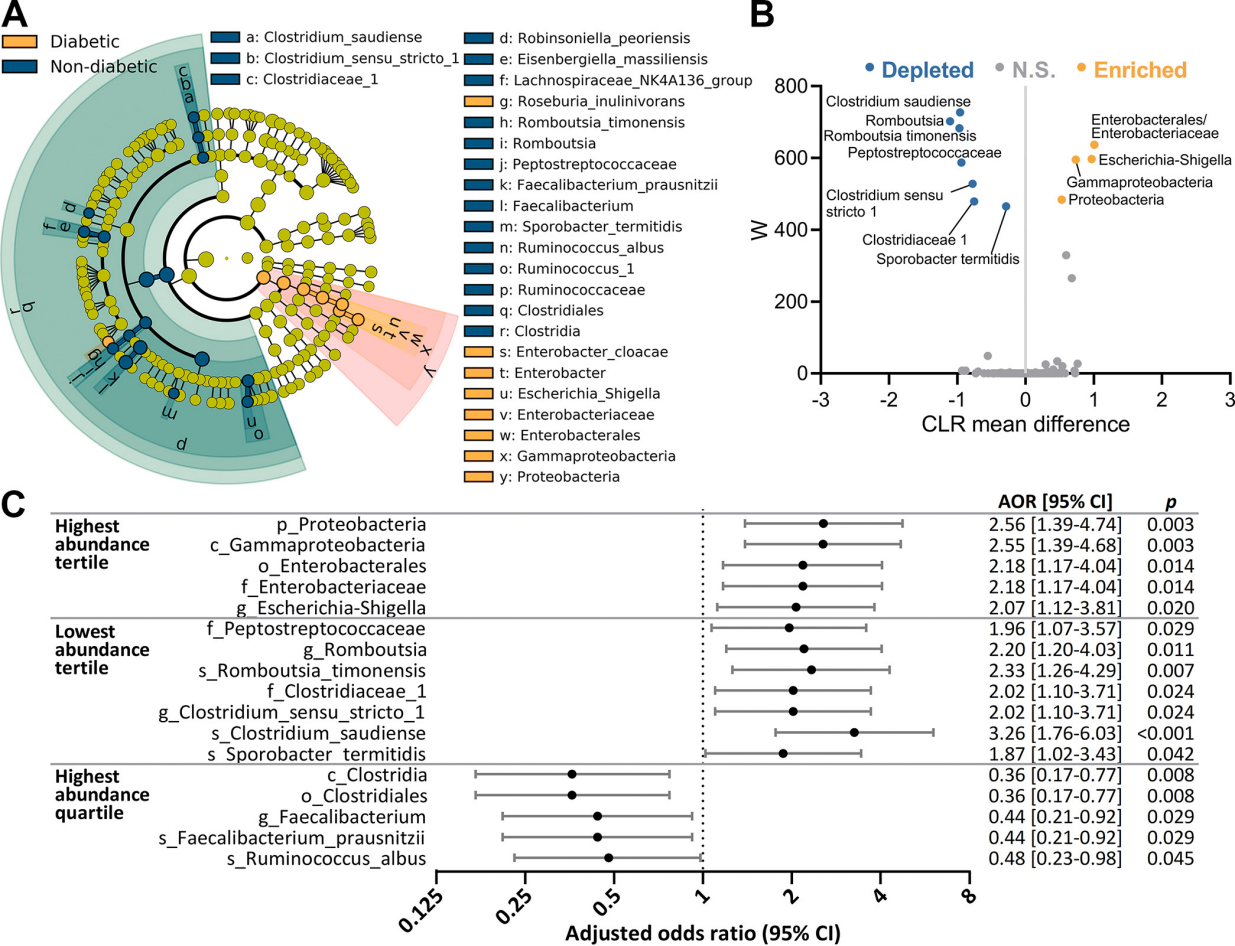
Bacterial taxa with altered abundance in subjects with diabetes. (A) Cladogram showing taxa with significantly different bacterial abundances between subjects with and without diabetes, as assessed by the linear discriminant analysis (LDA) effect size (LEfSe) algorithm. (B) Volcano plot of ANCOM analysis showing all bacterial taxa with ≥0.1% abundance in at least 25% of samples. Significance was determined using a false-discovery rate (FDR) of <0.05 and a *W* statistic above the 60th percentile. The *x* axis represents effect size based on the centered log ratio (CLR)-transformed mean difference in abundance between subjects with and without diabetes. Labels sharing a dot indicate taxa at different taxonomic levels, where all reads from the higher level are assigned to the taxa at the lower level. N.S., not significant. (C) Forest plot of significant associations of high and low bacterial abundance with diabetes. For bacteria enriched in subjects with diabetes, adjusted odds ratios (AORs) were calculated for diabetes in subjects with abundance in the highest tertile. For bacteria depleted in subjects with diabetes, AORs were calculated for diabetes in subjects with abundance in the lowest tertile and in subjects with abundance in the highest quartile. AOR, adjusted odds ratio (adjusted for age, gender, and body mass index [BMI]). Classifications at the phylum (p_), class (c_), order (o_), family (f_), genus (g_), and species (s_) levels are shown.

Twenty-five taxa with significant differences in abundance between subjects with and without diabetes were identified using linear discriminant analysis (LDA) effect size (LEfSe) analysis (Fig. 2A). Of these, 12 taxa were also considered significant by ANCOM analysis (false-discovery rate [FDR] < 0.05). Relative abundance and magnitude of change with diabetes for these 12 taxa are illustrated in Fig. S2 and Fig. 2B. Logistic regression analysis adjusting for age, gender, and BMI was additionally performed, validating these associations (Fig. 2C). Subjects with diabetes had significant enrichment of the *Proteobacteria* phylum (fold change [FC] = 1.6, adjusted odds ratio [AOR] = 2.56 [95% confidence intervals (CI) = 1.39 to 4.74], *P = *0.003), due to enrichment of the Gammaproteobacteria class, Enterobacterales order, and *Enterobacteriaceae* family. Within the *Enterobacteriaceae* family, there was enrichment of Escherichia*-Shigella* (FC = 3.2, AOR = 2.07 [95% CI = 1.12 to 3.81], *P = *0.020). Most of the taxa significantly depleted in diabetic subjects belonged to the *Clostridiales* order, with the strongest association with diabetes observed for the lowest-tertile abundance of Clostridium saudiense (FC = −17.2, AOR = 3.26 [95% CI = 1.76 to 6.03], *P < *0.001) and Romboutsia timonensis (FC = −5.5, AOR = 2.33 [95% CI = 1.26 to 4.29], *P = *0.007). A significantly reduced risk for diabetes was observed with the highest-quartile abundance of Clostridia and *Clostridiales* (FC = −1.1, AOR = 0.36 [95% CI = 0.17 to 0.77], *P = *0.008), Faecalibacterium and Faecalibacterium prausnitzii (FC = −1.2, AOR = 0.44 [95% CI = 0.21 to 0.92], *P = *0.029), and Ruminococcus albus (FC = −4.2, AOR = 0.48 [95% CI = 0.23 to 0.98], *P = *0.045).

10.1128/msystems.00033-22.2 FIG S2 Relative abundances of bacterial taxa with altered abundance in subjects with diabetes. Median relative abundances are shown for subjects with and without diabetes. Download FIG S2, TIF file, 2.3 MB. Copyright © 2022 Kwan et al. 2022 Kwan et al. https://creativecommons.org/licenses/by/4.0/ This content is distributed under the terms of the Creative Commons Attribution 4.0 International license.

### Potential contribution of genetics to high *Proteobacteria* abundance.

**FIG 3:**
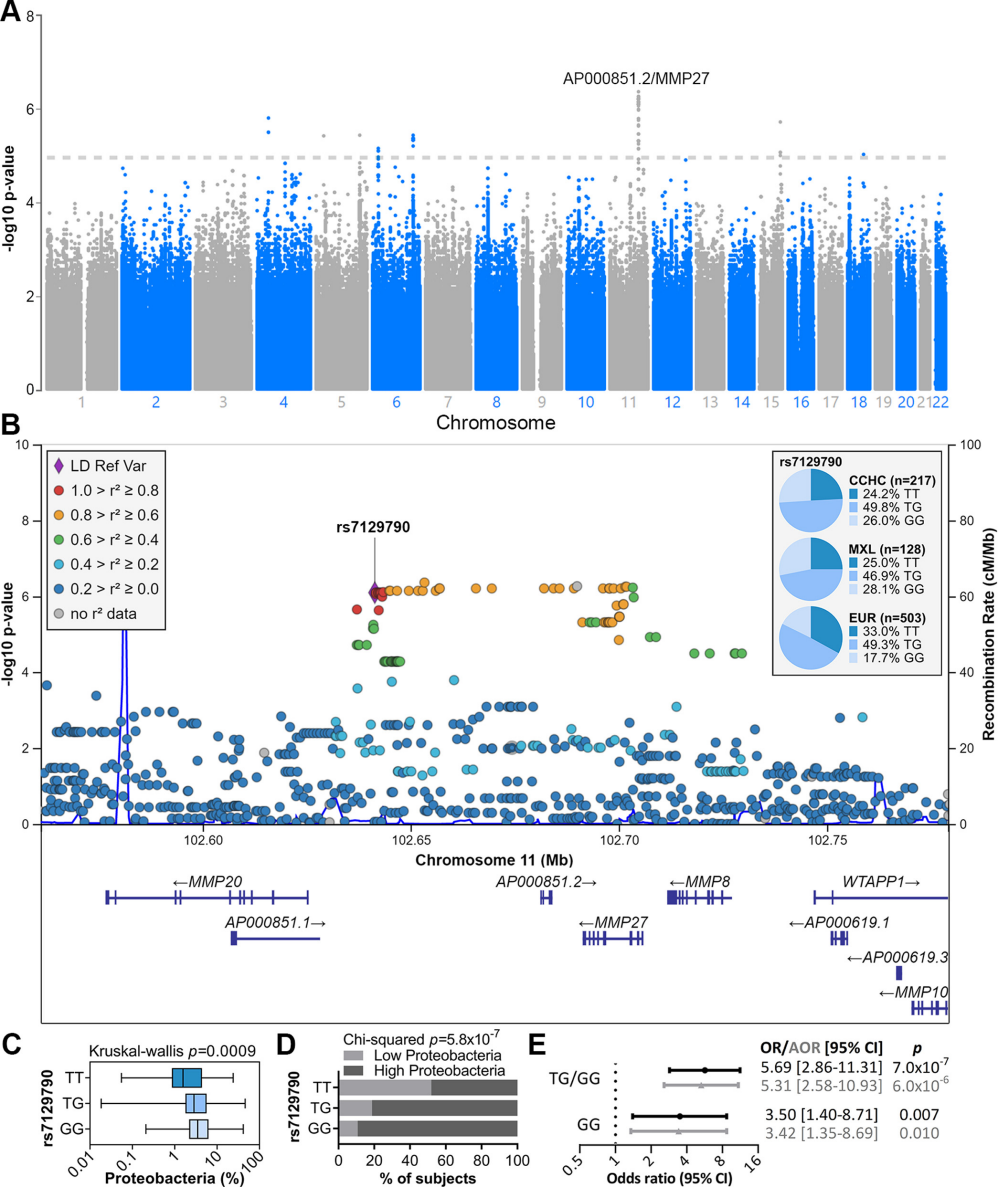
Host genetics associated with *Proteobacteria* abundance. (A) Manhattan plot for the genome-wide association study (GWAS) of high *Proteobacteria* abundance. (B) Regional association plot for the top locus associated with high *Proteobacteria* abundance. Genotype frequencies for rs7129790 are shown (MXL, subjects with Mexican ancestry in Los Angeles, CA; EUR, Europeans from the 1000 Genomes Project)]. (C) *Proteobacteria* abundance by rs7129790 genotype. Bars represent the median and interquartile range; error bars show the minimum and maximum abundances. (D) Percentage of subjects with high and low *Proteobacteria* abundance by rs7129790 genotype. (E) Forest plot showing the association between rs7129790 TG/GG and GG genotypes and high *Proteobacteria* abundance. AOR, adjusted odds ratio (adjusted for age, gender, and presence of diabetes).

To determine whether host genetics could influence *Proteobacteria* abundance, a genome-wide association study (GWAS) was performed with high *Proteobacteria* abundance as a dichotomized trait. A total of 139 subjects had genome-wide genotyping data available and were included in the GWAS. While no single-nucleotide polymorphism (SNP) displayed genome-wide significance (*P < *5 × 10^−8^), 89 SNPs were significantly associated with *Proteobacteria* abundance at the threshold of *P < *1 × 10^−5^ (Fig. 3A). The top 30 SNPs are shown in Table S1.

10.1128/msystems.00033-22.5 TABLE S1 Top 30 single-nucleotide polymorphisms (SNPs) from genome-wide association studies (GWAS) for high *Proteobacteria* abundance. A GWAS was performed on 139 Cameron County Hispanic Cohort (CCHC) subjects from the study cohort with genome-wide sequencing data. The 30 SNPs with the lowest *P* values are listed, sorted by chromosome and position. Odds ratios are for the minor allele relative to the major allele. Gene annotation was performed using SNPnexus v4. Functional annotation was performed using RegulomeDB v2.0 to derive probability scores and ranks. Chr, chromosome; MAF, minor allele frequency. Download Table S1, DOCX file, 0.02 MB. Copyright © 2022 Kwan et al. 2022 Kwan et al. https://creativecommons.org/licenses/by/4.0/ This content is distributed under the terms of the Creative Commons Attribution 4.0 International license.

All top 30 SNPs belonged to a locus on chromosome 11 near several matrix metalloproteinase (MMP) genes (*MMP20*, *MMP27*, *MMP8*, and *MMP10*) and long noncoding RNAs (Fig. 3B). Among them, rs7129790 (*P = *8.03 × 10^−7^) has a fair likelihood of having regulatory functions based on its RegulomeDB rank of 3a and probability score of 0.75. Based on expression quantitative trait loci (eQTL) data in the PhenoScanner database, the G allele is significantly associated with decreased expression of *MMP27* and *MMP8* in whole blood and increased expression of an uncharacterized gene transcript, ENSG00000255798.1, in small intestinal tissue. By PCR genotyping of subjects for which genome-wide genotyping data were not available, we confirmed that the G allele was significantly associated with high *Proteobacteria* abundance in the full set of 216 CCHC subjects (median abundances of 1.6%, 2.8%, and 3.5% for TT, TG, and GG, respectively; Kruskal-Wallis *P = *0.0009) (Fig. 3C and D). Logistic regression analysis further confirmed that the TG/GG and GG genotypes were significantly associated with high *Proteobacteria* abundance, even after adjusting for age, gender, and presence of diabetes (rs7129790-TG/GG: AOR = 5.31 [95% CI = 2.58 to 10.93], *P = *6.0 × 10^−6^; rs7129790-GG: AOR = 3.42 [95% CI = 1.35 to 8.69], *P = *0.010) (Fig. 3E). The frequency of the GG genotype was comparable between the CCHC and subjects with Mexican ancestry in Los Angeles (MXL) from the 1000 Genomes Project (26.1% and 28.1%), but higher than that in Europeans (EUR) from the 1000 Genomes Project (17.7%; *P = *0.011) (Fig. 3B).

### Metagenomic changes in diabetes.

**FIG 4:**
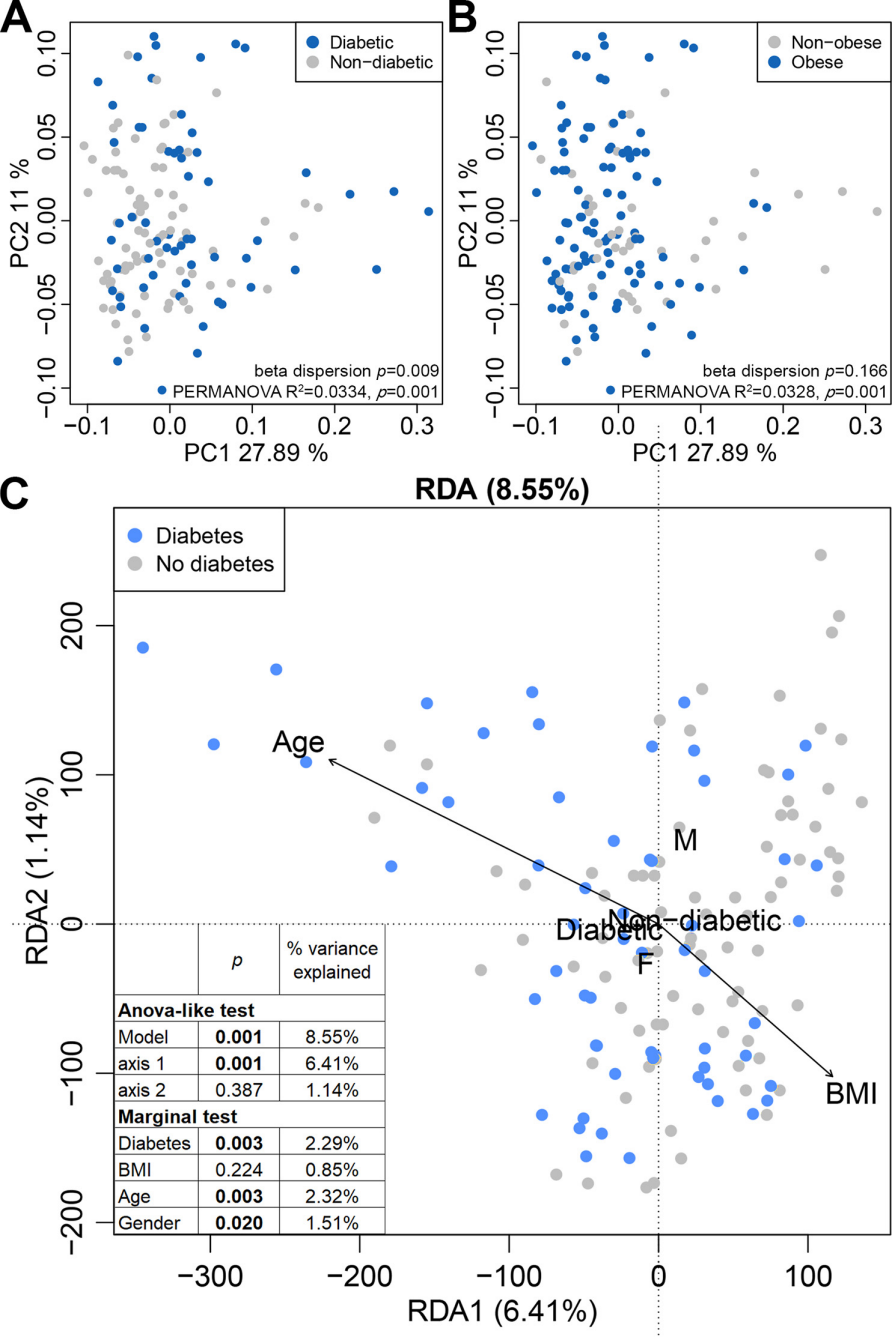
The stool metagenome profile is altered in diabetes. (A and B) PCoA plots of the 141 subjects for which whole-genome sequencing (WGS) was performed on stool samples, based on the Brays-Curtis distances of MetaCyc pathway abundance. Subjects were grouped by diabetes (A) and obesity statuses (B). (C) Redundancy analysis based on MetaCyc pathway abundance. Explanatory variables (clinical and demographic factors) are shown in black.

Finally, to identify microbiome functional changes associated with diabetes, shotgun metagenomic sequencing was performed on a subset of 141 CCHC study participants. Among them, 59 (41.8%) had diabetes. A significant shift in the overall metagenome profile was observed with diabetes (beta dispersion *P = *0.009, PERMANOVA *P = *0.001; 3.34% of variation explained) (Fig. 4A). A significant shift was also observed with obesity (beta dispersion *P = *0.166, PERMANOVA *P = *0.001; 3.28% of variation explained) (Fig. 4B). When we performed redundancy analysis of MetaCyc pathway abundances and using diabetes, BMI, age and gender as explanatory variables (Fig. 4C), diabetes remained significantly associated with metagenome profile changes (2.29% of variance explained; *P = *0.003).

**FIG 5:**
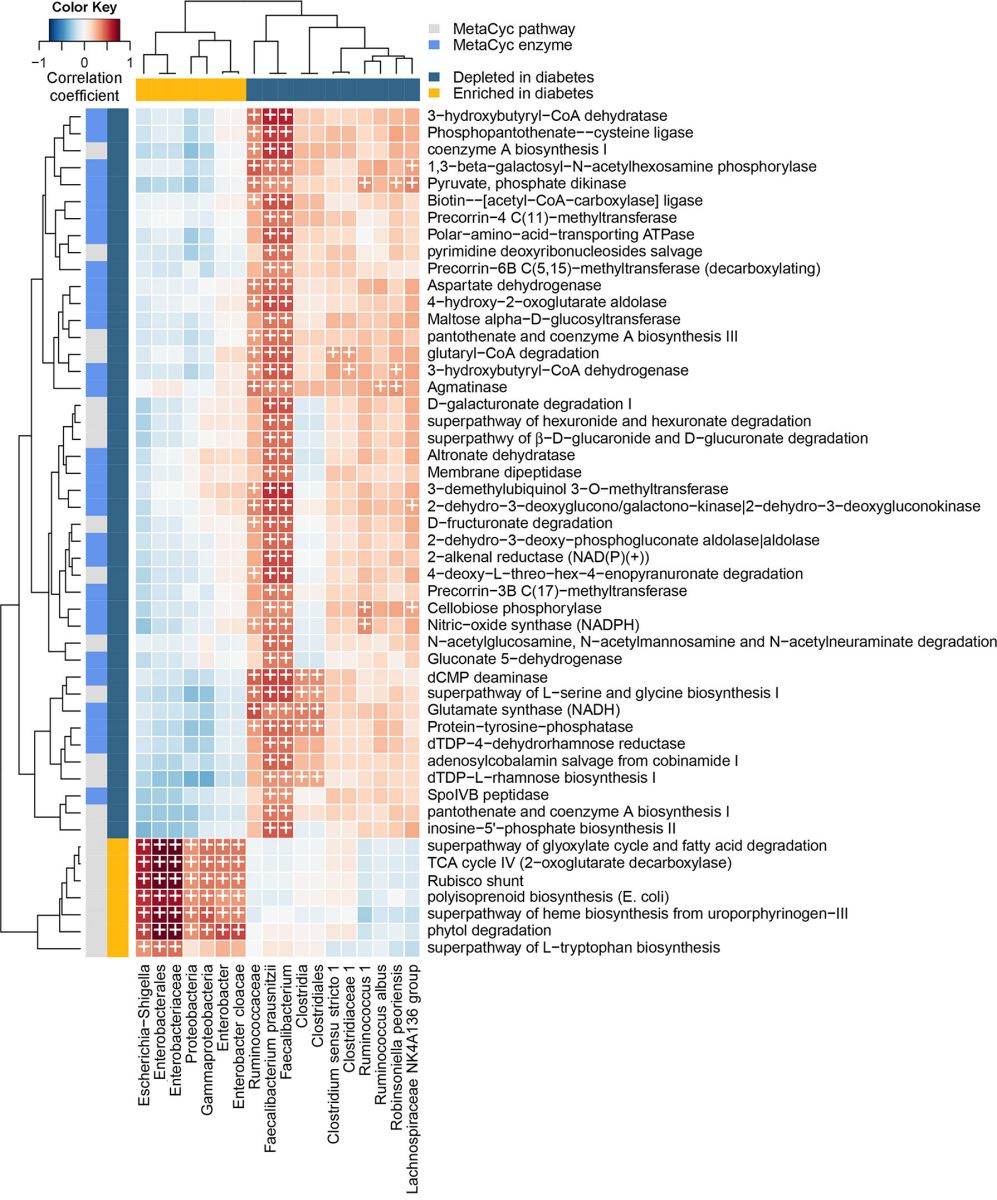
Correlation between bacterial abundance and stool metagenomic functions. Spearman’s correlation between bacterial taxa and MetaCyc pathways/enzymes with significantly altered abundance in diabetes. Rows represent pathways and enzymes; columns represent taxa. Only pathways/enzymes and taxa with at least one significant positive correlation (Benjamini-Hochberg-adjusted *P < *0.05 and *r_s_* ≥ 0.3, indicated with a cross symbol) are shown.

A total of 23 MetaCyc pathways and 32 MetaCyc reactions were significantly altered in subjects with diabetes (Fig. S3A and B). All significant reactions were depleted in subjects with diabetes. Correlation analysis between all diabetes-associated pathways and enzymes was performed (Fig. S4). Additionally, the significant positive correlations between pathways/enzymes and diabetes-associated bacteria are shown in Fig. 5. Many of the enriched pathways positively correlated with members of the *Proteobacteria* phylum, most strongly with the *Enterobacterales* order and the *Enterobacteriaceae* family. The strongest effect among pathways was observed for phytol degradation (FC = 3.10), with correlation coefficients (*r_s_*) with *Enterobacterales* and *Enterobacteriaceae* of 0.76 (*P < *0.001). There was also enrichment of the superpathway of heme biosynthesis from uroporphyrinogen-III (FC = 2.35) with *r_s_* correlation coefficients with *Enterobacterales* and *Enterobacteriaceae* of 0.74 (*P < *0.001). The superpathway of l-tryptophan biosynthesis was also enriched (FC = 2.31). On the other hand, there was depletion of pathways related to the biosynthesis of other amino acids (superpathway of l-serine and glycine biosynthesis I, FC = −1.18; l-isoleucine biosynthesis IV, FC = −1.30). Other depleted functions included highly intercorrelated pathways related to galacturonate and glucuronate catabolism (4-deoxy-l-threo-hex-4-enopyranuronate degradation, FC = −1.44; d-galacturonate degradation I, FC = −1.37; superpathway of hexuronide and hexuronate degradation, FC = −1.33; d-fructuronate degradation, FC = −1.33; superpathway of β-d-glucuronide and d-glucuronate degradation, FC = −1.31) (Fig. S3A and B and Fig. S4). The majority of the depleted MetaCyc pathways and enzymes were significantly correlated with depletion of *Faecalibacterium* and Faecalibacterium prausnitzii (Fig. 5).

10.1128/msystems.00033-22.3 FIG S3 MetaCyc pathways and enzymes with altered abundance in diabetes. (A and B) Significant differences in abundance of MetaCyc pathways and reactions were identified between subjects with and without diabetes. Significance was determined by ANCOM (false-discovery rate [FDR] of <0.05). Mean abundances for the differentially abundant pathways (A) and enzymes (B) performing the reactions are shown. Download FIG S3, TIF file, 0.5 MB. Copyright © 2022 Kwan et al. 2022 Kwan et al. https://creativecommons.org/licenses/by/4.0/ This content is distributed under the terms of the Creative Commons Attribution 4.0 International license.

10.1128/msystems.00033-22.4 FIG S4 Correlation matrix between stool metagenomic functions significantly associated with diabetes. Spearman’s correlation matrix analysis was performed between all pathways and enzymes from Fig. S3A and B. The cross symbol indicates a significant correlation (*P < *0.05 and |*r_s_*| ≥ 0.3). Download FIG S4, TIF file, 1.6 MB. Copyright © 2022 Kwan et al. 2022 Kwan et al. https://creativecommons.org/licenses/by/4.0/ This content is distributed under the terms of the Creative Commons Attribution 4.0 International license.

## DISCUSSION

In this study, we aimed to determine the association between the gut microbiome and diabetes, which disproportionately affects Mexican Americans in South Texas. The study was performed on subjects from the CCHC, a population-based cohort of Mexican Americans in South Texas recruited from households that have high prevalences of obesity, diabetes, and NAFLD. The microbiome profiles of Mexican Americans from CCHC without metabolic diseases were distinct from those of healthy Caucasians in Texas, but also from those of Hispanics living in California, with significant enrichment of *Firmicutes* and *Actinobacteria* and depletion of *Bacteroidetes*, confirming that both region and ethnicity impact the overall microbiome composition of this population. While the *Bacteroidetes* phylum was depleted overall, members within this phylum, Prevotellaceae and Prevotella copri were enriched, in agreement with previous reports (12). It is, however, important to mention that careful interpretation of the differences in diversity and microbiome profiles between groups is needed due to the small sample sizes of both healthy Texans and Hispanics in California, as well as possible batch effects arising from differences in sample collection and processing between the AGP and the CCHC.

The presence of diabetes was associated with widespread depletion across the Clostridia class and enrichment of the *Enterobacteriaceae* family in the *Proteobacteria* phylum, namely, Enterobacter cloacae and Escherichia*-*Shigella, which are considered opportunistic pathogens (13–15). Members of the depleted *Clostridiales* order, including Faecalibacterium prausnitzii, *Peptostreptococcaceae*, and *Clostridium sensu stricto* 1, are known producers of butyrate (16, 17), which contributes to intestinal barrier integrity, attenuates chronic inflammation through promotion of regulatory T cells, and protects against proliferation of pathogens (18). Loss of these butyrate-producing bacteria may contribute to the overgrowth of lipopolysaccharide-expressing Gram-negative members of the *Proteobacteria* phylum, which subsequently activate Toll-like receptor 4 (TLR4) signaling to induce chronic low-grade inflammation. These observations are in concordance with other studies on gut microbiome changes in type 2 diabetes. The abundance of Faecalibacterium prausnitzii has consistently shown a negative association with diabetes in studies where it was reported (19). Furthermore, its abundance increased after weight loss in patients with type 2 diabetes, suggesting that high BMI may contribute to its depletion (20). Depletion of butyrate-producing bacteria and enrichment of opportunistic pathogens was observed in a Chinese cohort (21). Similarly, using a multicountry cohort of Danish, Swedish, and Chinese subjects, Forslund et al. reported a depletion of butyrate-producing *Clostridiales* species (22). Finally, higher abundances of Peptostreptococcaceae, Romboutsia, and *Clostridium sensu stricto* 1 were associated with reduced risk of type 2 diabetes in two large Dutch population-based cohorts (23). The authors also reported a significant association between gut microbiome variation and insulin resistance as measured by HOMA score. However, while HOMA scores were significantly different between subjects with and without diabetes in our study, we did not observe a significant association between gut microbiome variation and HOMA scores (PERMANOVA *P = *0.098; 0.82% of variation explained), indicating that insulin resistance was not a major confounder of our findings.

Host genetics contribute to gut microbiome variation (24). We identified a locus on chromosome 11 near several *MMP* genes, where the minor allele G for rs7129790 was associated with high *Proteobacteria* abundance. This SNP is associated with decreased expression levels of *MMP27* and *MMP8* in whole blood, and increased expression of the gene transcript ENSG00000255798.1 in the small intestine. *MMP8* is primarily expressed by polymorphonuclear neutrophils (PMNs) and is involved in their chemotaxis. Impaired infiltration of PMNs to the site of lipopolysaccharide stimulation has been observed in *MMP8*-null mice (25). PMNs are involved in homeostasis of the intestinal mucosa via elimination of pathogenic bacteria (26). Therefore, decreased expression of *MMP8* may have implications for PMN recruitment to the intestinal mucosa and for their ability to maintain gut homeostasis. The function of *MMP27* has not been well elucidated, but expression was found to be enriched in immunoglobulin G (IgG)/IgM-stimulated B cells (27). The majority of activated B cells differentiate into plasma cells that produce secretory IgA, which maintains gut homeostasis by coating specific bacterial species to attenuate bacterial invasion and inflammatory responses (28). Mice lacking IgA exhibited persistent expansion of *Proteobacteria* and exaggerated inflammation (29). Furthermore, unclassified *Enterobacteriaceae* were enriched in humans with IgA deficiency (30). Therefore, rs7129790 may affect the ability of the innate and adaptive immune systems to maintain gut homeostasis due to decreased expression of *MMP8* and *MMP27*. The minor allele frequency for rs7129790 was significantly higher in the CCHC study cohort compared to that in Europeans, suggesting that this variant may contribute to differences in disease susceptibility between ethnicities. The top SNPs in our GWAS were not found in previous microbiome GWAS studies, which may be due to the lack of ethnic diversity in GWAS studies, with the majority of participants being of European ancestry (24, 31).

Finally, shotgun metagenomic sequencing revealed an important shift in functional profiles in subjects with diabetes. This may be due to the widespread enrichment of *Proteobacteria* in diabetic patients. A previous study concluded that the abundance of most genes in the microbiome are invariable across individual hosts, while a minor subset exhibits significant variability. Furthermore, the authors observed that *Proteobacteria* were significantly enriched for variable genes, thus contributing to interindividual variations in the metagenome (more so than other phyla) (32). The functional changes observed in subjects with diabetes suggest dysregulated amino acid metabolism, namely, increased biosynthesis of l-tryptophan and decreased biosynthesis of l-serine, glycine, and l-isoleucine. Circulating levels of l-tryptophan have been linked to increased insulin resistance and risk of diabetes (33). Conversely, l-serine and glycine are associated with improved insulin sensitivity (34, 35). There was also depletion of multiple pathways and enzymes related to galacturonate and glucuronate catabolism. d-Galacturonate is the main component of pectin, a complex plant polysaccharide that is abundant in fruits and vegetables and indigestible by human enzymes. Fermentation by gut bacteria gives rise to various metabolites, including butyrate (36). Related MetaCyc pathways and enzymes were among those significantly correlated with Faecalibacterium prausnitzii, which is known to degrade pectin (37). Subjects with diabetes also had enrichment of a heme biosynthesis superpathway, which correlated strongly with the enrichment of *Enterobacteriaceae*. Heme is an iron-containing cofactor that is required by bacterial pathogens for essential functions and virulence (38, 39). High dietary heme iron intake has also been associated in multiple prospective studies with increased risk of type 2 diabetes due to the production of reactive oxygen species under excess iron conditions (40).

In conclusion, we identified changes in the gut microbiome associated with diabetes in Mexican Americans of South Texas, which suggested promotion of a chronic proinflammatory environment, loss of butyrate production, and compromised intestinal barrier integrity. This taxonomic shift was accompanied by significant changes in the metagenome, which indicated dysregulation of amino acid metabolism, reduced galacturonate and glucuronate catabolism, and increased heme biosynthesis. Integrative analysis of the metagenomic changes with stool metatranscriptomics and blood metabolomics would be highly valuable. Similarly, validation of microbiome-based risk models for diabetes should be evaluated in prospective cohort studies.

## MATERIALS AND METHODS

### Demographic and laboratory data collection for study participants.

The study included 216 participants from the CCHC (41). We excluded subjects who had antibiotic, probiotic, or proton pump inhibitor use within 30 days of stool collection. Written informed consent was obtained from each participant, and the study protocol was approved by the Committee for the Protection of Human Subjects of participating institutions. Fasting blood samples were collected and analyzed for metabolic and lipid panels. Homeostatic model assessment (HOMA) scores were calculated using the formula: glucose (mg/dL)/18 × insulin (mU/L)/22.5. Categorical or diagnostic definitions were described by the following criteria: obesity (BMI ≥ 30), diabetes (fasting blood glucose ≥ 126 mg/dL, HbA1c ≥ 6.5%, history of diabetic medication or diagnosed with diabetes), elevated aspartate aminotransferase (AST) (> 33 U/L), elevated alanine aminotransferase (ALT) (>40 U/L for men and >31 U/L for women), heavy drinking (alcohol consumption of >20 g/day for men and >10 g/day for women), moderate drinking (nonzero weekly consumption that did not reach heavy drinking criteria), former smoking (lifetime consumption of ≥100 cigarettes plus no smoking at time of survey), current smoking (lifetime consumption of ≥100 cigarettes plus smoking at time of survey). Demographic and laboratory parameters of the study participants are described in Table 1.

### Stool DNA extraction, 16S rRNA gene amplicon sequencing, and bioinformatic analysis.

Stool samples were collected from all 216 participants using the Omnigene stool kit and analyzed for 16S sequencing at the MD Anderson Cancer Center Microbiome Core Facility. Bacterial genomic DNA was extracted using the QIAamp Fast DNA stool minikit (Qiagen). The V4 region of the bacterial 16S rRNA gene was amplified by PCR (forward primer, 5′-AATGATACGGCGACCACCGAGATCTACACGCTXXXXXXXXXXXXTATGGTAATTGTGTGYCAGCMGCCGCGGTAA-3′, where XXXXXXXXXXXX is an index sequence for multiplexing libraries; reverse primer, 5′-CAAGCAGAAGACGGCATACGAGATAGTCAGCCAGCCGGACTACNVGGGTWTCTAAT-3′). Libraries were purified using Zymo I-96 column purification and analyzed on the 4200 TapeStation system (Agilent). Barcoded amplicons were pooled in equal concentrations. Pooled libraries were quantified by Qubit fluorometer, and the molarity was calculated based on amplicon size. Sequencing (250-bp paired end) was performed on the Illumina MiSeq platform (Read1 seq primer, 5′-TATGGTAATTGTGTGYCAGCMGCCGCGGTAA-3′; Read2 seq primer, 5′-AGTCAGCCAGCCGGACTACNVGGGTWTCTAAT-3′; and primer, AATGATACGGCGACCACCGAGATCTACACGCT). Paired-end reads were demultiplexed and split in QIIME 1. Merging of paired-end reads to create consensus sequences was done by VSEARCH v7, allowing up to a maximum of 10 mismatches. The “cluster_otus” command, an implementation of the UPARSE algorithm, was used to perform 97% related operational taxonomic units (OTU) clustering. Denoising was done by the “unoise3” command. OTUs were then subjected to taxonomy assignment using Mothur with the Silva database (v132). The number of 16S sequence reads was not significantly different between stool samples from nondiabetic and diabetic subjects (mean [range] of 91,125 [25,663 to 226,058] versus 89,300 [30,904 to 183,829]; *P = *0.651).

### Acquisition and processing of data from the American Gut Project.

FASTQ files for selected samples from the American Gut Project (AGP) were downloaded from the European Bioinformatics Institute website (BioProject accession number PRJEB11419) (42, 43). Subjects with antibiotic use within the prior year, cancer, liver disease, obesity, or diabetes were excluded. For comparison with the CCHC Hispanic population of South Texas, we included 31 CCHC subjects that were not heavy drinkers and did not have obesity, diabetes, abnormal AST levels, or abnormal ALT levels. AGP samples were run through the same pipeline as that for CCHC samples. As AGP samples were collected without preservatives, OTUs corresponding to “blooming” genera previously identified in AGP samples (44) were removed from all samples. These included Citrobacter, Enterobacter, Escherichia-Shigella, Klebsiella, Morganella, and Pseudomonas. Relative abundances of taxa were generated after removal of blooms. In addition, samples with fewer than 2,500 sequence reads were excluded from analysis. Alpha diversity of samples from the CCHC and AGP was estimated using QIIME 2 from a randomly rarefied data set of 2,500 reads per sample with 10 iterations.

### Functional profiling of stool samples by shotgun metagenomic sequencing.

Shotgun metagenomic sequencing was performed (CosmosID, Inc., Rockville, MD) to a sequencing depth of 12 million reads (±20%), on stool samples from 141 of the 216 participants. DNA was isolated using the DNeasy PowerSoil Pro kit (Qiagen) and quantified by Qubit fluorometer (Thermo Fisher). DNA libraries were prepared using the Illumina Nextera XT library preparation kit. Libraries were assessed with a Qubit fluorometer (Thermo Fisher) and sequenced on an Illumina HiSeq platform using 150-bp paired-end sequencing. The percentage of sequencing reads aligned to the human genome was determined to be minimal (mean of 0.04%) via Bowtie2 (v2.4.1) (45), using GRCh38 and major single-nucleotide polymorphisms (SNPs) as the reference genome, with default Bowtie2 parameters. Initial quality control, adapter trimming, and preprocessing of metagenomic sequencing reads were performed using bbduk (https://jgi.doe.gov/data-and-tools/bbtools/). Quality-controlled reads were subjected to a translated search using Diamond against a comprehensive and nonredundant protein sequence database, UniRef90. UniRef90 represents a clustering of all nonredundant protein sequences in UniProt, such that each sequence in a cluster aligns with 90% identity and 80% coverage of the longest sequence in the cluster. The mapping of metagenomic reads to gene sequences was weighted by mapping quality, coverage, and gene sequence length to estimate community-wide weighted gene family abundances, which were subsequently annotated to MetaCyc reactions (Metabolic Enzymes) to reconstruct and quantify MetaCyc metabolic pathways as described previously (46). Abundance values were normalized using total-sum scaling normalization to produce copies per million.

### Genome-wide association study and PCR genotyping.

GWAS was performed on 139 of the 216 CCHC participants with whole-genome-imputed SNP genotypes. Genome-wide genotyping was performed using the Illumina Multi-Ethnic Genotyping Array (MEGA) with 2.7 million SNPs, optimized for the Hispanic population. After stringent preimputation quality control measures, including SNP/subject-wise genotyping missing rate, Hardy-Weinberg equilibrium, heterozygosity rate, sample duplication, and sex inconsistency, we imputed the GWAS data to the TOPMed whole-genome sequencing reference panel using the Michigan Imputation Server (47). The R package GENESIS (48, 49) was used to perform GWAS of 9.3 million SNPs with a minor allele frequency ≥3% and imputation score (*R*^2^) of >0.4. The GENESIS analysis pipeline explicitly models population structure, relatedness between individuals, and ancestry admixture. GWAS was performed for *Proteobacteria*, after dichotomization into high/low abundance based on the cutoff used for logistic regression (first quartile [Q1]). As no SNPs passed the genome-wide significance threshold of *P < *5 × 10^−8^, we used *P < *1 × 10^−5^ for suggestive significance. Gene annotation was performed with SNPnexus v4 (50). The likelihood of each SNP having regulatory functions was predicted using RegulomeDB v2.0 (51). The PhenoScanner v2 database (52) was used to access previous reports of associated gene expression in expression quantitative trait loci (eQTL) studies (*P < *1 × 10^−5^).

rs7129790 was additionally genotyped by PCR in the CCHC study participants for which genome-wide genotyping data were not available, using predesigned TaqMan SNP human genotyping assays (Thermo Fisher), the SsoAdvanced Universal Probes Supermix (Bio-Rad), and the Applied Biosystems ViiA7 real-time PCR system (Thermo Fisher). Results were analyzed using QuantStudio real-time PCR Software v1.3 (Thermo Fisher). For analysis of selected SNPs in the full data set, we considered a *P* value of <0.05 to indicate significance for all statistical tests (Chi-squared, Kruskal-Wallis, and logistic regression).

### Statistical analyses.

Statistical analyses were performed in R (version 4.0.0; R Foundation for Statistical Computing, Vienna, Austria). Principal-coordinate analysis (PCoA) was performed using the “cmdscale” function and the weighted UniFrac distances of the OTU tables. Beta dispersion and PERMANOVA tests, using weighted UniFrac distances, were performed with the vegan package. Differences in bacterial abundance were assessed using the linear discriminant analysis (LDA) effect size (LefSe) tool (53), with a *P* value of <0.05 and a log_10_ LDA score of >2 considered significant. Taxa with ≥0.1% abundance in at least 25% of samples were included. Additional differential abundance analysis of taxa was performed with ANCOM v2.1 (54), where a false-discovery rate (FDR) significance threshold of 0.05 was used for calculation of *W* statistics. *W* statistics greater than or equal to the 60th percentile of the *W* distribution were considered significant. Logistic regression was performed using the “glm.fit” function to obtain adjusted odds ratios (AORs) and 95% confidence intervals (CI). For bacteria enriched in subjects with diabetes, AORs were calculated for diabetes in subjects with abundance in the highest tertile. For bacteria depleted in subjects with diabetes, AORs were calculated for diabetes in subjects with abundance in the lowest tertile. AORs were also calculated with abundance in the highest quartile. Pairwise correlations were performed using Spearman’s correlation in R, with *P* values adjusted for multiple testing using the Benjamini-Hochberg method. For metagenomic functional data, PCoA plots, PERMANOVA tests, and redundancy analysis (RDA) were performed using Brays-Curtis distances based on MetaCyc pathway abundances. Differences in MetaCyc pathways and reactions were assessed by ANCOM. Pathways and reactions with ≥0.01% abundance in at least 25% of samples were included.

### Data availability.

The accompanying 16S rRNA sequencing data have been deposited into the Sequence Read Archive (SRA) of the National Center for Biotechnology Information (NCBI) under BioProject accession number PRJNA734860.
