# Supplementary figures and images for: Gut Microbiome Alterations Associated with Diabetes in Mexican Americans in South Texas
Source: mSystems. 2022 Apr 28;7(3):e00033-22. doi: 10.1128/msystems.00033-22 (PMC9238400; doi:10.1128/msystems.00033-22)

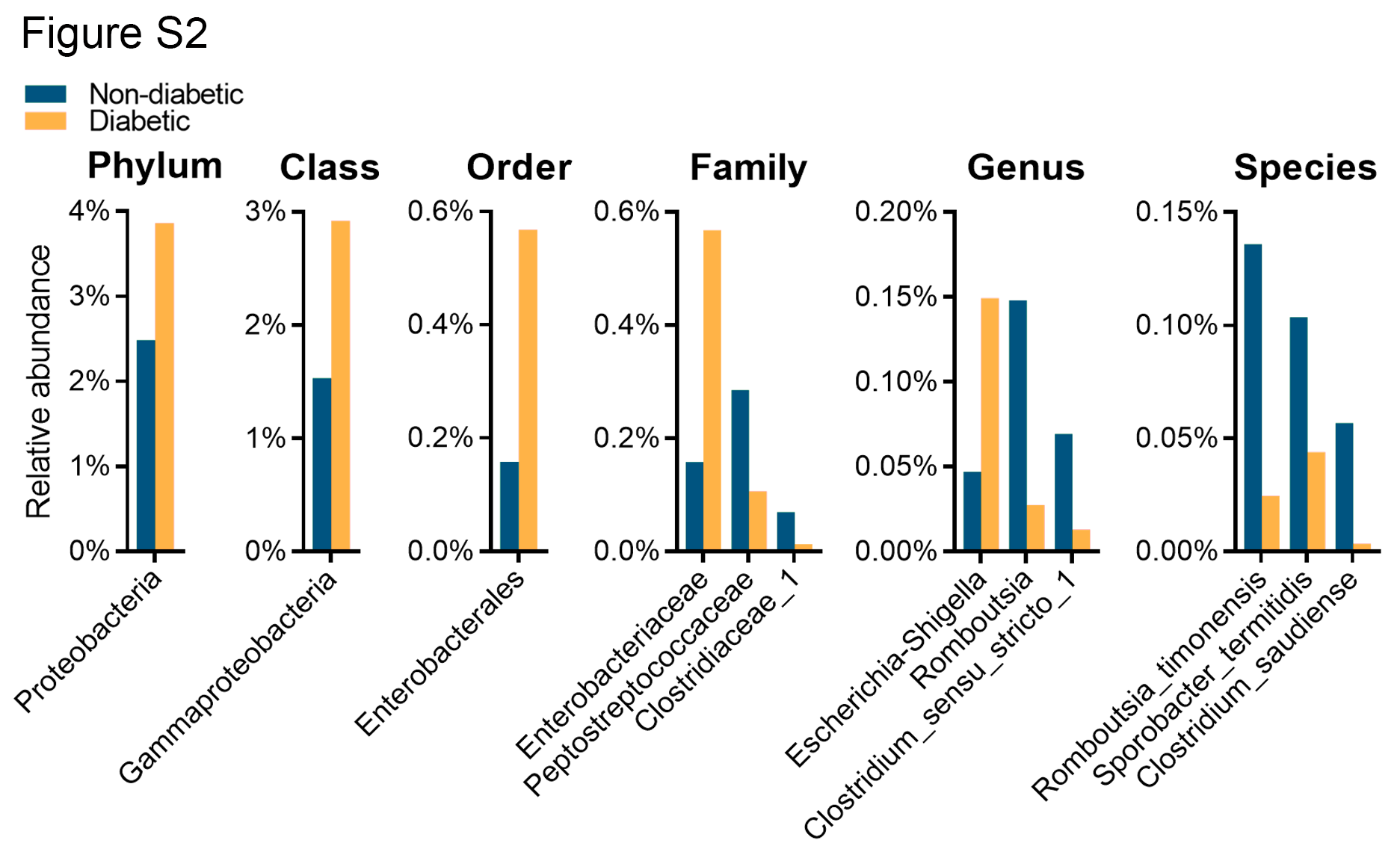

Supplement: FIG S2 [file msystems.00033-22-s0002.tif]

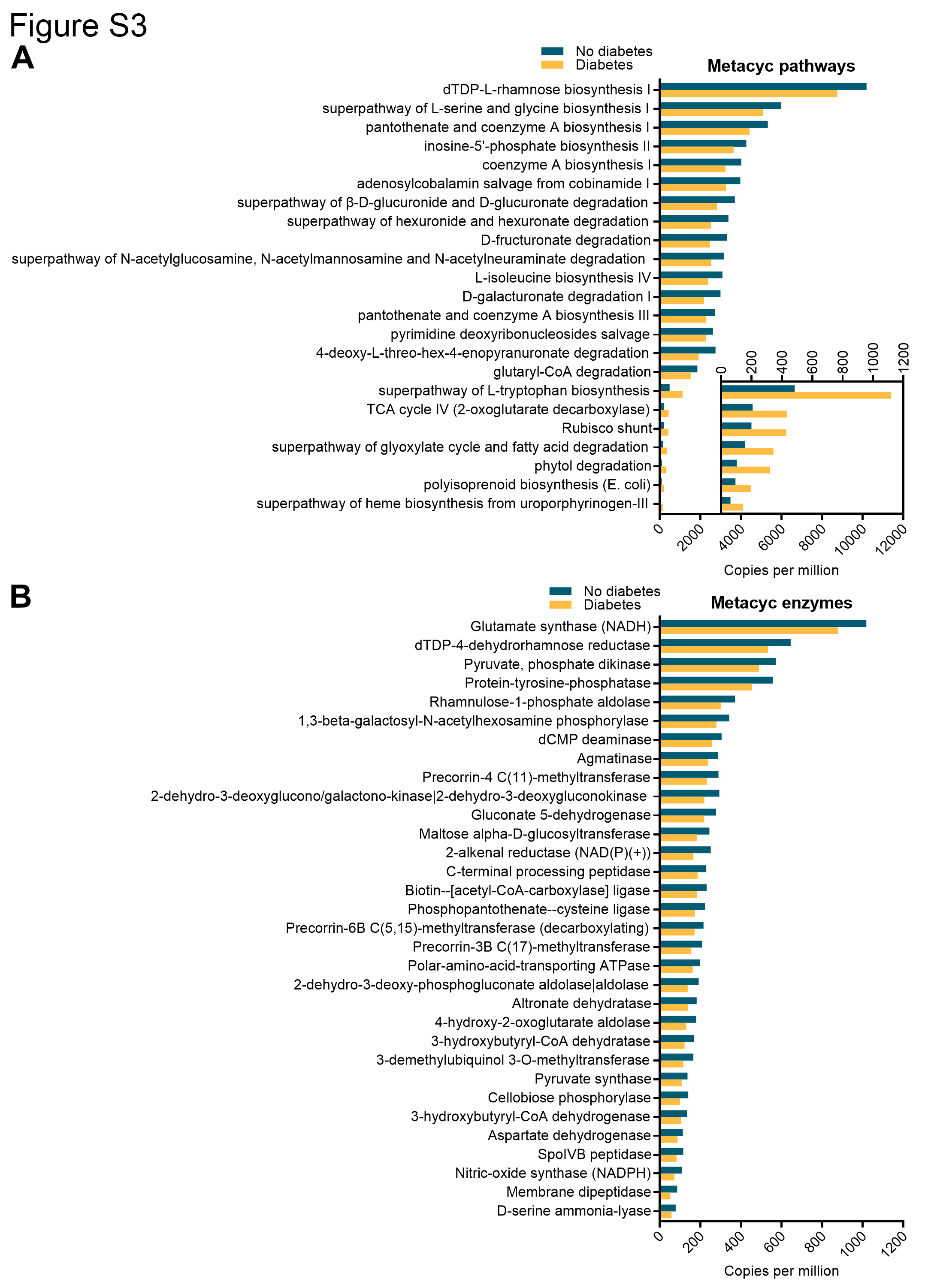

Supplement: FIG S3 [file msystems.00033-22-s0003.tif]

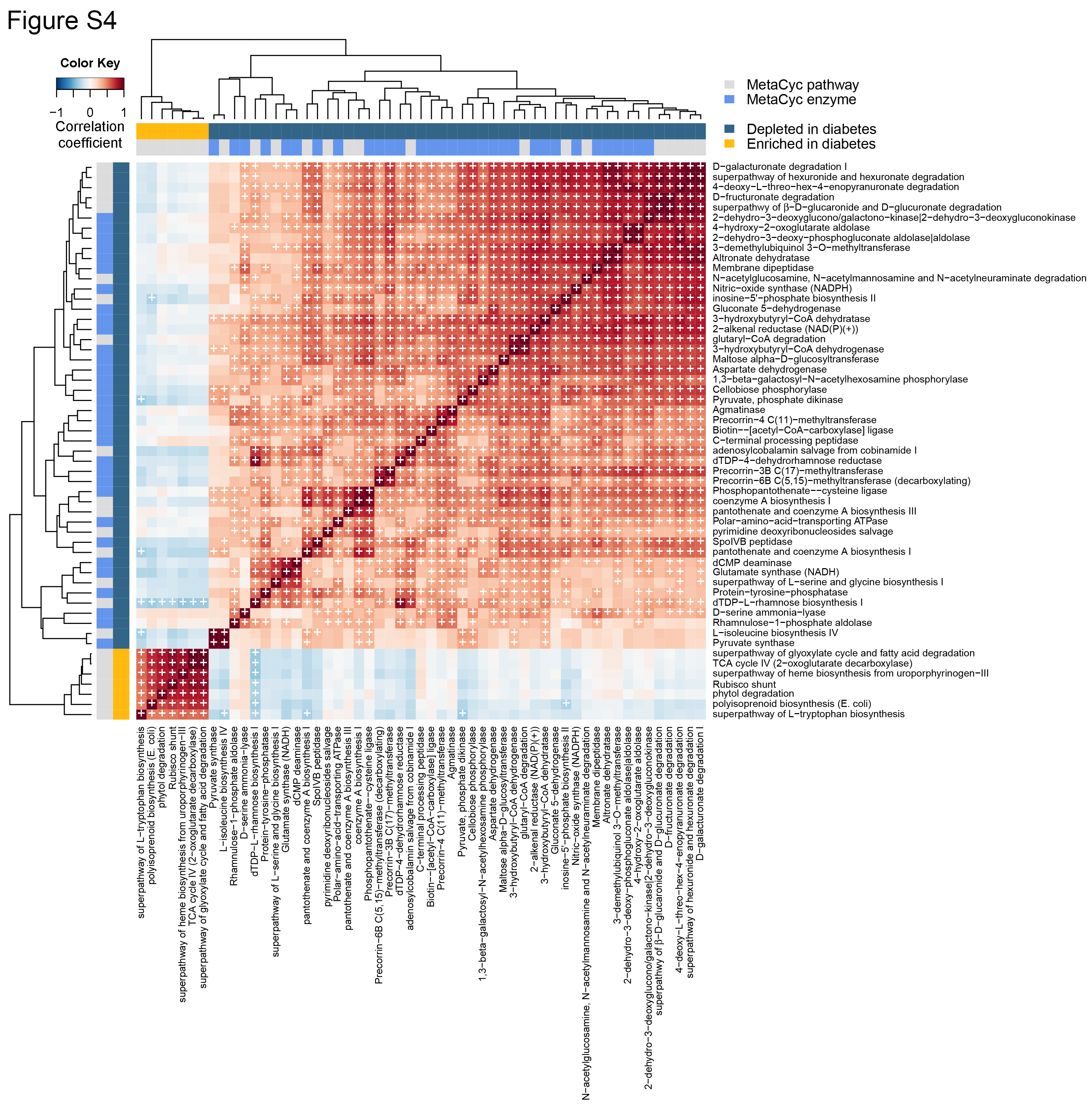

Supplement: FIG S4 [file msystems.00033-22-s0004.tif]
