# Supplementary material for: Gut Microbiome Alterations Associated with Diabetes in Mexican Americans in South Texas
Source: mSystems. 2022 Apr 28;7(3):e00033-22. doi: 10.1128/msystems.00033-22 (PMC9238400; doi:10.1128/msystems.00033-22)
Supplement: TABLE S1 [file msystems.00033-22-s0005.docx]

**Supplemental Table S1**

| **SNP** | **Chr** | **Position (hg38)** | **Major allele** | **Minor allele** | **MAF** | ***p*** | **Odds ratio (95% CI)** | **Overlapped Gene** | **Nearest Upstream Gene** | **Nearest Downstream Gene** | **Regulome DB score** | **Regulome DB rank** |
| --- | --- | --- | --- | --- | --- | --- | --- | --- | --- | --- | --- | --- |
| rs7109950 | 11 | 102641320 | G | A | 0.475 | 8.03E-07 | 4.623 (2.517-8.492) | None | AP000851.1 | AP000851.2 | 0.6091 | 4 |
| rs7129777 | 11 | 102641355 | T | C | 0.475 | 8.03E-07 | 4.623 (2.517-8.492) | None | AP000851.1 | AP000851.2 | 0.5069 | 3a |
| rs7129790 | 11 | 102641383 | T | G | 0.475 | 8.03E-07 | 4.623 (2.517-8.492) | None | AP000851.1 | AP000851.2 | 0.7541 | 3a |
| rs10160697 | 11 | 102641623 | A | C | 0.475 | 8.03E-07 | 4.623 (2.517-8.492) | None | AP000851.1 | AP000851.2 | 0.5896 | 5 |
| rs4754862 | 11 | 102643427 | A | G | 0.475 | 7.75E-07 | 4.79 (2.573-8.916) | None | AP000851.1 | AP000851.2 | 0.1345 | 5 |
| rs2509029 | 11 | 102644853 | C | T | 0.583 | 7.17E-07 | 5.092 (2.675-9.692) | None | AP000851.1 | AP000851.2 | 0.1841 | 7 |
| rs7113003 | 11 | 102645083 | A | G | 0.583 | 7.17E-07 | 5.092 (2.675-9.692) | None | AP000851.1 | AP000851.2 | 0.1841 | 7 |
| rs2846711 | 11 | 102646733 | G | C | 0.583 | 7.17E-07 | 5.092 (2.675-9.692) | None | AP000851.1 | AP000851.2 | 0.1345 | 5 |
| rs1276281 | 11 | 102649563 | T | C | 0.583 | 7.17E-07 | 5.092 (2.675-9.692) | None | AP000851.1 | AP000851.2 | 0.0067 | 6 |
| rs1276279 | 11 | 102651776 | T | C | 0.583 | 7.17E-07 | 5.092 (2.675-9.692) | None | AP000851.1 | AP000851.2 | 0.1841 | 7 |
| rs1276278 | 11 | 102652910 | G | A | 0.583 | 7.17E-07 | 5.092 (2.675-9.692) | None | AP000851.1 | AP000851.2 | 0.1841 | 7 |
| rs34473819 | 11 | 102653313 | TA | T | 0.586 | 4.35E-07 | 5.183 (2.738-9.811) | None | AP000851.1 | AP000851.2 | 0.1109 | 6 |
| rs1940041 | 11 | 102656034 | C | G | 0.583 | 7.17E-07 | 5.092 (2.675-9.692) | None | AP000851.1 | AP000851.2 | 0.5896 | 5 |
| rs2846715 | 11 | 102656873 | A | G | 0.586 | 6.22E-07 | 5.096 (2.686-9.668) | None | AP000851.1 | AP000851.2 | 0.0000 | 6 |
| rs2846342 | 11 | 102656989 | C | T | 0.586 | 6.22E-07 | 5.096 (2.686-9.668) | None | AP000851.1 | AP000851.2 | 0.1841 | 7 |
| rs1276257 | 11 | 102665683 | G | A | 0.586 | 6.22E-07 | 5.096 (2.686-9.668) | None | AP000851.1 | AP000851.2 | 0.1345 | 5 |
| rs1940473 | 11 | 102669499 | T | C | 0.586 | 6.22E-07 | 5.096 (2.686-9.668) | None | AP000851.1 | AP000851.2 | 0.6777 | 5 |
| rs1298740 | 11 | 102682048 | G | C | 0.586 | 6.22E-07 | 5.096 (2.686-9.668) | AP000851.2 | None | None | 0.1345 | 5 |
| rs1276275 | 11 | 102684347 | A | T | 0.586 | 6.22E-07 | 5.096 (2.686-9.668) | None | AP000851.2 | MMP27 | 0.9816 | 5 |
| rs1276274 | 11 | 102685781 | T | C | 0.586 | 6.22E-07 | 5.096 (2.686-9.668) | None | AP000851.2 | MMP27 | 0.1345 | 5 |
| rs10791590 | 11 | 102689231 | G | T | 0.586 | 6.22E-07 | 5.096 (2.686-9.668) | None | AP000851.2 | MMP27 | 0.5047 | 6 |
| rs200028103 | 11 | 102689987 | CTTT  TGTT  TTG | C | 0.410 | 5.44E-07 | 0.196 (0.103-0.371) | None | AP000851.2 | MMP27 | 0.1763 | 5 |
| rs1296594 | 11 | 102696212 | G | T | 0.586 | 6.22E-07 | 5.096 (2.686-9.668) | MMP27 | None | None | 0.1841 | 7 |
| rs2464363 | 11 | 102697733 | A | G | 0.586 | 6.22E-07 | 5.096 (2.686-9.668) | MMP27 | None | None | 0.0050 | 6 |
| rs2155555 | 11 | 102697795 | T | C | 0.586 | 6.22E-07 | 5.096 (2.686-9.668) | MMP27 | None | None | 0.1841 | 7 |
| rs2846724 | 11 | 102698772 | C | T | 0.586 | 6.22E-07 | 5.096 (2.686-9.668) | MMP27 | None | None | 0.1345 | 5 |
| rs2701992 | 11 | 102700500 | C | T | 0.414 | 6.22E-07 | 0.196 (0.103-0.372) | MMP27 | None | None | 0.4327 | 6 |
| rs2846704 | 11 | 102701587 | A | G | 0.507 | 5.60E-07 | 0.203 (0.109-0.380) | MMP27 | None | None | 0.1841 | 7 |
| rs2701993 | 11 | 102701889 | G | A | 0.507 | 5.60E-07 | 0.203 (0.109-0.380) | MMP27 | None | None | 0.1841 | 7 |
| rs2846705 | 11 | 102703377 | C | T | 0.439 | 5.92E-07 | 0.191 (0.100-0.366) | MMP27 | None | None | 0.1131 | 6 |
